# Characterization of SARS-CoV-2 Omicron spike RBD reveals significantly decreased stability, severe evasion of neutralizing-antibody recognition but unaffected engagement by decoy ACE2 modified for enhanced RBD binding

**DOI:** 10.1038/s41392-022-00914-2

**Published:** 2022-02-21

**Authors:** Sheng Lin, Zimin Chen, Xindan Zhang, Ao Wen, Xin Yuan, Chongzhang Yu, Jing Yang, Bin He, Yu Cao, Guangwen Lu

**Affiliations:** 1 West China Hospital Emergency Department (WCHED), State Key Laboratory of Biotherapy, West China Hospital, Sichuan University, 610041 Chengdu, Sichuan China; 2 Disaster Medicine Center, West China Hospital, Sichuan University, 610041 Chengdu, Sichuan China

**Keywords:** Vaccines, Infectious diseases

**Dear Editor,**

The recent-emerging Omicron variant (B.1.1.529 lineage) of severe acute respiratory syndrome coronavirus 2 (SARS-CoV-2) has raised serious public concern because of its rapid regional- and global-transmission. As of 11th January 2022, the Omicron variant has spread to 140 countries, territories or areas through infected air travelers, and the number is continuously increasing.^1^ Currently, Omicron has outcompeted the Delta variant (B.1.617.2 lineage) in many countries (e.g., USA, United Kingdom, France, Italy, etc.), becoming the dominant circulating variant and causing surges in weekly infections.^1^ Therefore, it is an urgent issue to re-evaluate and/or re-develop effective agents to combat the potential Omicron pandemic.

The Omicron variant accumulated unusual large number of mutations (over 60 amino-acid substitutions/deletions/insertions) in its genome-encoded proteins. Among these proteins, the surface-located spike (S) that determines viral infectivity and antigenicity, carries 30 amino-acid substitutions, 6 residue deletions, and 3 residue insertions. Most importantly, the receptor-binding domain in spike (S-RBD), which is the main target for therapeutic antibodies and the key component of prophylactic vaccines, harbors 15 substitutions, including G339D, S371L, S373P, S375F, K417N, N440K, G446S, S477N, T478K, E484A, Q493R, G496S, Q498R, N501Y, and Y505H. Most of the substitutions are near or located on the human angiotensin-converting enzyme 2 (ACE2) binding interface, and all of the substitutions could be mapped to one or more of the known antigenic sites in S-RBD (Fig. 1a), suggesting that S-RBD of the Omicron variant might behave differently from that of the original SARS-CoV-2 strain when interacting with the ACE2 receptor and the therapeutic antibodies.

**Fig. 1:**
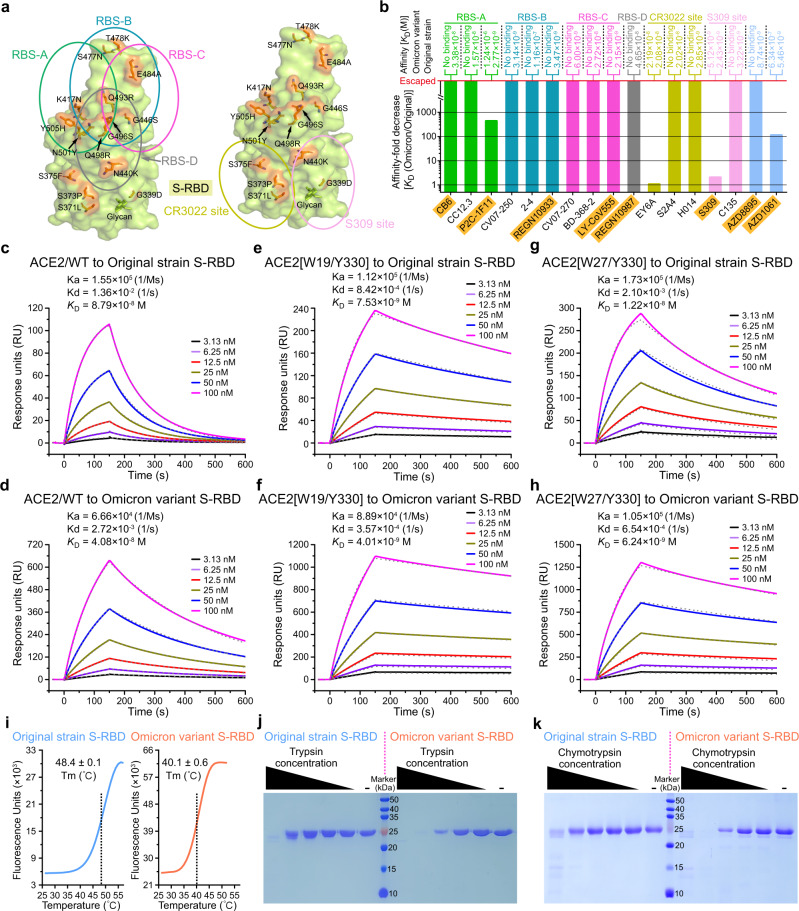
Antibody-escape profile, receptor-binding capacity, and biochemical property of Omicron variant S-RBD. **a** Multiple previously identified antigenic sites were mapped on original strain S-RBD (PDB code: 6XC4). The RBS-A, RBS-B, RBS-C, RBS-D were circled on left panel, the CR3022 site and S309 site were circled on right panel. Amino-acid mutations of Omicron variant S-RBD were marked on both panels. **b** The binding affinities between SARS-CoV-2 S-RBD (original strain and Omicron variant) and each representative antibody (in scFv form) calculated by SPR. The dissociation constant (*K*_D_) values and associated affinity-fold decrease [*K*_D_ (Omicron/Original] were individually shown. The antibodies that have been approved for clinical use were highlighted by shadowing in orange. The related real-time binding profiles were demonstrated in Supplementary Fig. S2. **c**–**h** The interaction between SARS-CoV-2 S-RBD (original strain and Omicron variant) and ACE2 proteins [wild-type or affinity-enhanced ACE2 mutants] characterized by SPR. The real-time binding profiles and calculated kinetic parameters are shown. **i** A DSF assay characterizing the thermostability of original strain and Omicron variant S-RBDs. The fluorescence-unit curve and melting temperature (Tm) for each S-RBD were shown. **j**, **k** Protease-digestion assays with fivefold serially diluted Trypsin (**j**) or Chymotrypsin (**k**) towards original strain S-RBD and Omicron variant S-RBD

In order to evaluate the impact of the Omicron mutations, we targeted the multiple previously identified antigenic sites in S-RBD [including RBS-A, RBS-B, RBS-C, RBS-D, CR3022 site, and S309 site (Fig. 1a)],^2^ selected a series of representative neutralizing antibodies for each site (CB6, CC12.3 and P2C-1F11 for RBS-A, CV07-250, 2-4 and REGN10933 for RBS-B, CV07-270, BD-368-2 and LY-CoV555 for RBS-C, REGN10987 for RBD-D, EY6A, S2A4 and H014 for CR3022 site, S309 and C135 for S309 site), and prepared the antibody protein in the single-chain variable fragment (scFv) form by in-vitro refolding method (Supplementary Fig. S1a). For each antibody, its affinities towards SARS-CoV-2 S-RBDs of the original strain and the Omicron variant (Supplementary Fig. S1b) were individually determined via surface plasmon resonance (SPR) for quantitative comparison of the binding capacity difference. Expectedly, all the antibodies tested readily bound to original strain S-RBD, showing nano-molar affinities (Fig. 1b and Supplementary Fig. S2). Towards Omicron S-RBD, however, only EY6A targeting CR3022 site and S309 targeting S309 site retained comparable binding. The remaining antibodies, especially those targeting the RBS-A, -B, -C and -D sites, were either inert in S-RBD recognition or showed significantly reduced binding capacity (decreased >400 folds), demonstrating significant escape of neutralizing-antibody recognition for Omicron S-RBD. It is notable that of the antibodies tested, CB6 (Etesevimab), LY-CoV555 (Bamlanivimab), P2C-1F11 (Amubarvimab), REGN10933 (Casirivimab), REGN10987 (Imdevimab), and S309 (Sotrovimab) have been approved for clinical use. We also evaluated another pair of clinically used antibodies [AZD8895 (Tixagevimab) and AZD1061 (Cilgavimab)], which also showed severely impaired binding towards Omicron S-RBD (Fig. 1b and Supplementary Fig. S2). These results raised concerns over their therapeutic efficacies against the Omicron virus. Noted that the binding of S309 to S-RBD was only marginally affected by the Omicron-specific mutations, we believe this clinically approved antibody should remain effective in combating Omicron.

Considering that the Omicron variant has a potential to cause another global SARS-CoV-2 pandemic, evaluation of the binding capacity between Omicron variant S-RBD and its receptor ACE2 has become another focus. Thus, we subsequently side-by-side determined the binding affinities of original strain S-RBD and Omicron variant S-RBD to human ACE2 (Supplementary Fig. S1c). With SPR, the kinetic-affinity values were determined to be 87.9 nM for original strain S-RBD and 40.8 nM for Omicron variant S-RBD, respectively (Fig. 1c, d). These values highlight ~2.2-fold-enhanced receptor-binding with Omicron S-RBD. In light of the previous report showing that soluble ectodomain protein of ACE2 could function as a “neutralizing decoy” to block SARS-CoV-2 entry,^3^ we thus further measured the binding capability between Omicron S-RBD and our affinity-enhanced ACE2 decoy proteins (ACE2[W19/Y330] and ACE2[W27/Y330]). As expected, towards the original strain and Omicron variant S-RBDs, ACE2[W19/Y330] and ACE2[W27/Y330] both showed apparently higher binding capacities than wild-type ACE2 (ACE2/WT) (Fig. 1e–h). The calculated affinities revealed ~10.2-fold increase for ACE2[W19/Y330] and ~6.5-fold increase for ACE2[W27/Y330] towards Omicron S-RBD, respectively. These observations highlighted that the improved binding between SARS-CoV-2 S-RBD and the affinity-enhanced decoy ACE2s were not affected by the Omicron mutations, forming stark contrast to the tested antibodies, which showed high degree of immune evasion. Since the safety of recombinant ACE2 have been verified in humans,^4^ the affinity-enhanced decoy ACE2s might be developed as weapons to fight against the Omicron variant and/or other emerging SARS-CoV-2 variants that use human ACE2 as the functional receptor.

As SARS-CoV-2 S-RBD is the most important component in vaccine preparation, we thus further evaluated the thermal stability and protease-digestion sensitivity of the Omicron variant S-RBD protein. The melting temperatures (Tms) of both SARS-CoV-2 original strain and Omicron variant S-RBDs were determined in parallel using differential scanning fluorimetry (DSF). Unexpectedly, the calculated Tm value for the Omicron variant S-RBD protein was significantly lower than that of the original strain S-RBD (decreased by ~8.3 °C) (Fig. 1i), demonstrating impaired protein thermal stability due to the mutations in Omicron S-RBD. We also performed the incomplete-digestion assay using trypsin and chymotrypsin to investigate the susceptibility of both S-RBDs to protease digestion. In comparison to S-RBD of the original strain, its Omicron counterpart was indeed much more easily digested by both proteases (Fig. 1j, k). Such observation was well consistent with the introduction of several basic amino acids (N440K, T478K, Q493R, Q498R, and Y505H for trypsin digestion) and aromatic/hydrophobic residues (S371L, S375F, and N501Y for chymotrypsin digestion) in the Omicron S-RBD primary amino-acid sequence. The results also suggested that more attention should be paid to Omicron S-RBD to prevent its potential denaturation and/or degradation when developing RBD-based Omicron vaccines. In addition, it has been shown that the SARS-CoV-2 virus could infect individuals through the digestive tracts, and further cause symptoms in the situs.^5^ Since both trypsin and chymotrypsin are a class of digestion-related proteases, they are very common and abundant in human digestive tracts. The decreased thermal stability and increased digestion susceptibility observed with Omicron S-RBD seem to coincide with the less virulence of the Omicron virus, at least along the digestive tract.

In conclusion, our study revealed that the multiple mutations on Omicron S-RBD have significantly compromised antibody recognition. Nevertheless, the Omicron-specific mutations did not affect S-RBD binding by affinity-enhanced decoy ACE2 proteins, highlighting future development of decoy ACE2 and its affinity-enhanced proteins for potential treatment of the Omicron infections.

## Supplementary information

Supplementary Materials

## Data Availability

The datasets used and/or analyzed during this study are available from the corresponding author on reasonable request.
