# Supplementary material for: Characterization of SARS-CoV-2 Omicron spike RBD reveals significantly decreased stability, severe evasion of neutralizing-antibody recognition but unaffected engagement by decoy ACE2 modified for enhanced RBD binding
Source: Signal Transduct Target Ther. 2022 Feb 21;7:56. doi: 10.1038/s41392-022-00914-2 (PMC8860268; doi:10.1038/s41392-022-00914-2)
Supplement: Supplementary file 1 — Supplementary Materials [file 41392_2022_914_MOESM1_ESM.docx]

Supplementary Materials for

**Characterization of SARS-CoV-2 Omicron spike RBD reveals significantly decreased stability, severe evasion of neutralizing-antibody recognition but unaffected engagement by decoy ACE2 modified for enhanced RBD binding**

Sheng Lin^1#^, Zimin Chen^1#^, Xindan Zhang^1^, Ao Wen^1^, Xin Yuan^1^, Chongzhang Yu^1^, Jing Yang^1^, Bin He^1^, Yu Cao^1,2^, Guangwen Lu^1*^

^1^ West China Hospital Emergency Department (WCHED), State Key Laboratory of Biotherapy, West China Hospital, Sichuan University, 610041, Chengdu, Sichuan, China

^2^ Disaster Medicine Center, West China Hospital, Sichuan University, 610041, Chengdu, Sichuan, China

^#^ These authors contributed equally: Sheng Lin, Zimin Chen

* Correspondence: Guangwen Lu ([lugw@scu.edu.cn](mailto:lugw@scu.edu.cn))

**This file includes:**

Materials and Methods

Supplementary Fig. S1 and S2

**Materials and Methods**

**Cloning, expression and purification**

For each scFv preparation, the variable regions of the light chain (VL) and heavy chain (VH) were joined via a (Gly-Gly-Gly-Gly-Ser)_4_ linker, and the protein was expressed as inclusion body in *E. coli* and further refolded *in vitro*. In brief, the coding sequence of each representative antibody, including CB6, CC12.3, P2C-1F11, CV07-250, 2-4, REGN10933, CV07-270, BD-368-2, LY-CoV555, REGN10987, EY6A, S2A4, H014, S309, C135, AZD8895 or AZD1061 was synthesized in scFv form (GENERAL BIOL Corporation) and sub-cloned into pET-21a vector. The resultant recombinant plasmids were transformed into *E. coli* BL21 (DE3) for expression. The cells containing plasmids were then grown in LB medium supplemented with 100 µg/ml ampicillin at 37°C and induced with 400 µM isopropyl-β-D-thiogalactopyranoside (IPTG) at 37°C for about 6 hours. Inclusion bodies were then extracted and refolded as previously described^1^. Briefly, aliquots of inclusion bodies were diluted dropwise into a stirring refolding buffer consisting of 100 mM Tris-HCl (pH 8.0), 400 mM L-Arg HCl, 2 mM EDTA, 5 mM reduced glutathione and 0.5 mM oxidized glutathione, and incubated overnight to complete the refolding process. Subsequently, the refolded protein was concentrated using an Amicon Stirred Cell concentrator with a 10 kDa cutoff membrane and then adjusted to size-exclusion chromatography (SEC) buffer containing 10 mM HEPES-NaOH (pH 7.5) and 150 mM NaCl. The refolded scFvs were then further purified by gel filtration chromatography on a Superdex 75 Increase 10/300 GL column (GE Healthcare).

The proteins for SARS-CoV-2 original strain S-RBD (residues 320-537 in spike protein), Omicron variant (B.1.1.529 lineage) S-RBD (residues 320-537 in spike protein, bearing G339D, S371L, S373P, S375F, K417N, N440K, G446S, S477N, T478K, E484A, Q493R, G496S, Q498R, N501Y and Y505H mutations), human wild-type ACE2 (ACE2/WT), and the affinity-enhanced human ACE2 mutants (ACE2[W19/Y330] and ACE2[W27/Y330]) were expressed and purified as previously described^2^. Finally, these proteins were purified by gel filtration chromatography in SEC buffer by using Superdex 75 Increase 10/300 GL column.

**Surface plasmon resonance (SPR) assay**

All the SPR experiments were performed with the BIAcore 8K system (GE Healthcare). SARS-CoV-2 S-RBDs (original strain and Omicron variant) were individually immobilized onto the CM5 sensor chip (GE Healthcare) using the Amine Coupling Kit (GE Healthcare). Gradient concentrations of scFv protein (CB6, CC12.3, P2C-1F11, CV07-250, 2-4, REGN10933, CV07-270, BD-368-2, LY-CoV555, REGN10987, EY6A, S2A4, H014, S309, C135, AZD8895 or AZD1061) or ACE2 proteins (ACE2/WT, ACE2[W19/Y330] or ACE2[W27/Y330]) were flowed over S-RBD in the running buffer containing 10 mM HEPES-NaOH (pH 7.5), 150 mM NaCl and 0.05% Tween-20 at a rate of 30 μl/min. The obtained kinetic data were further analyzed with the Biacore Insight Evaluation Software (GE Healthcare) by using the 1:1 (Langmuir) binding model.

**Differential scanning fluorimetry (DSF) assay**

The DSF assay was carried out as previously described^3^. SYPRO Orange dye (Sigma) was used to probe protein thermal denaturation. For the DSF assay, 20 µl of the sample (original strain S-RBD or Omicron variant S-RBD, at a protein concentration of 10 μM in SEC buffer) was heated using a linear temperature-gradient of 25-95°C in 75 min using CFX Connect Real-Time System (Bio-Rad). The fluorescence signal as a function of temperature was monitored continuously. Each sample was measured in triplet and fitted with the Boltzmann equation using GraphPad Prism 6 (GraphPad Software).

**Protease-digestion assay**

Trypsin and chymotrypsin were used as the digestive protease and the system was conducted in the buffer containing 50 mM HEPES-NaOH (pH 7.5) and 150 mM NaCl. 400 µg/ml substrate sample (original strain S-RBD or Omicron variant S-RBD) was in parallel digested by 5-fold serially diluted trypsin or chymotrypsin protease (ranging from 300 µg/ml to 0.48 µg/ml). After a 30-min incubation at 37°C, the reaction was quenched by the addition of 1 mM PMSF. The product sample was finally analyzed on SDS-PAGE gel and visualized by staining with Coomassie blue.

**References:**

1. Zhang, S. et al. Competition of cell adhesion and immune recognition: insights into the interaction between CRTAM and nectin-like 2. *Structure* **21**, 1430-1439 (2013).

2. Ye, F. et al. S19W, T27W, and N330Y mutations in ACE2 enhance SARS-CoV-2 S-RBD binding toward both wild-type and antibody-resistant viruses and its molecular basis. *Signal Transduct Target Ther* **6**, 343 (2021).

3. Lin, S. et al. Crystal structure of SARS-CoV-2 nsp10/nsp16 2'-O-methylase and its implication on antiviral drug design. *Signal Transduct Target Ther* **5**, 131 (2020).

Supplementary Fig. S1


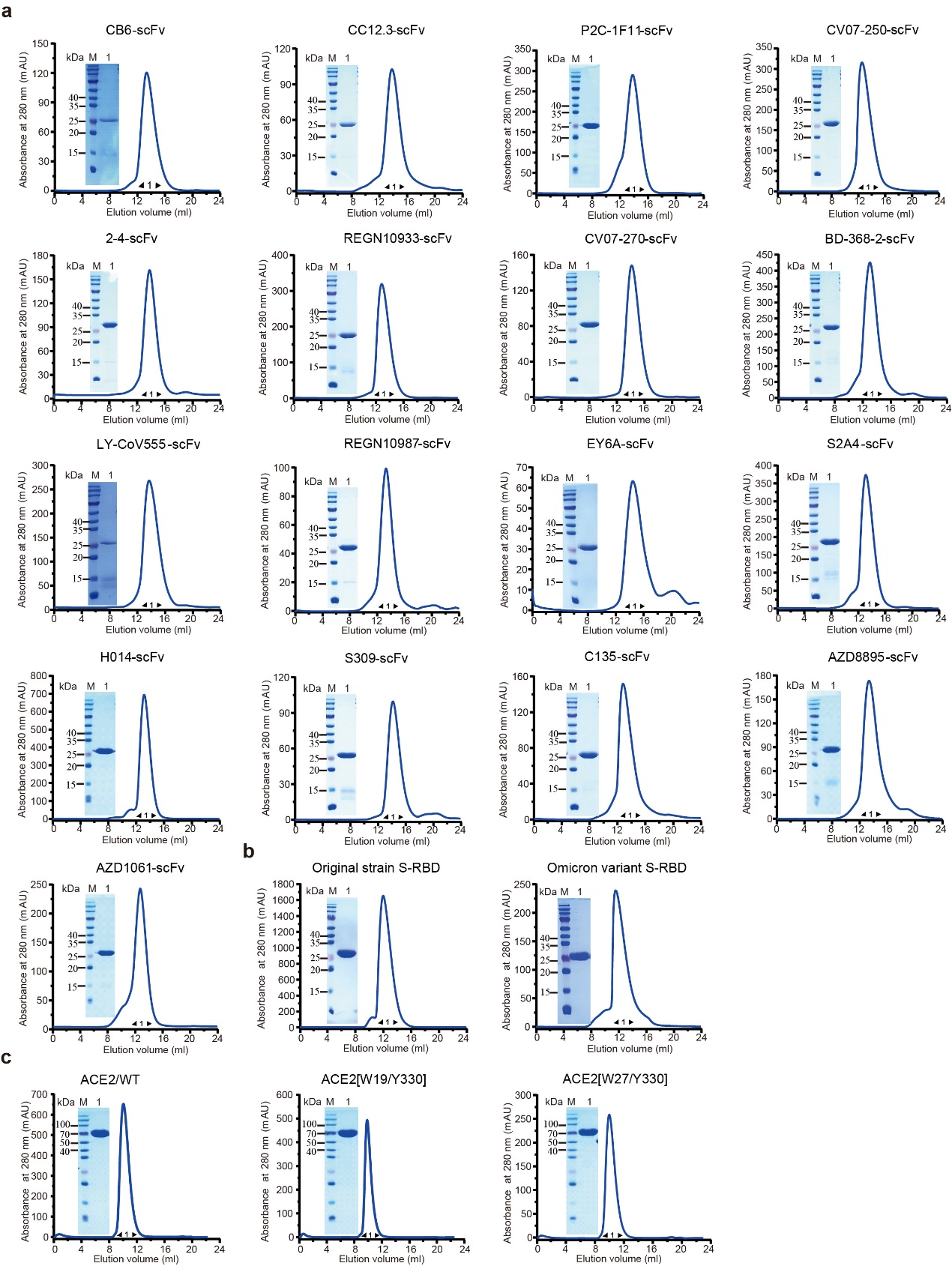


**Supplementary Fig. S1. Solution behavior of each protein used in this study on a Superdex 75 Increase 10/300 GL column.** The representative 280-nm absorbance curves and the SDS-PAGE migration profiles of the proteins are shown. **a** Antibodies (CB6, CC12.3, P2C-1F11, CV07-250, 2-4, REGN10933, CV07-270, BD-368-2, LY-CoV555, REGN10987, EY6A, S2A4, H014, S309, C135, AZD8895, and AZD1061) in scFv forms. **b** SARS-CoV-2 S-RBD proteins (original strain and Omicron variant). **c** ACE2 proteins (ACE2/WT: wild-type ACE2; ACE2[W19/Y330]: ACE2 carrying the S19W, N330Y, H374A, H378A, and E402A mutations; ACE2[W27/Y330]: ACE2 harboring the T27W, N330Y, H374A, H378A, and E402A mutations).

Supplementary Fig. S2


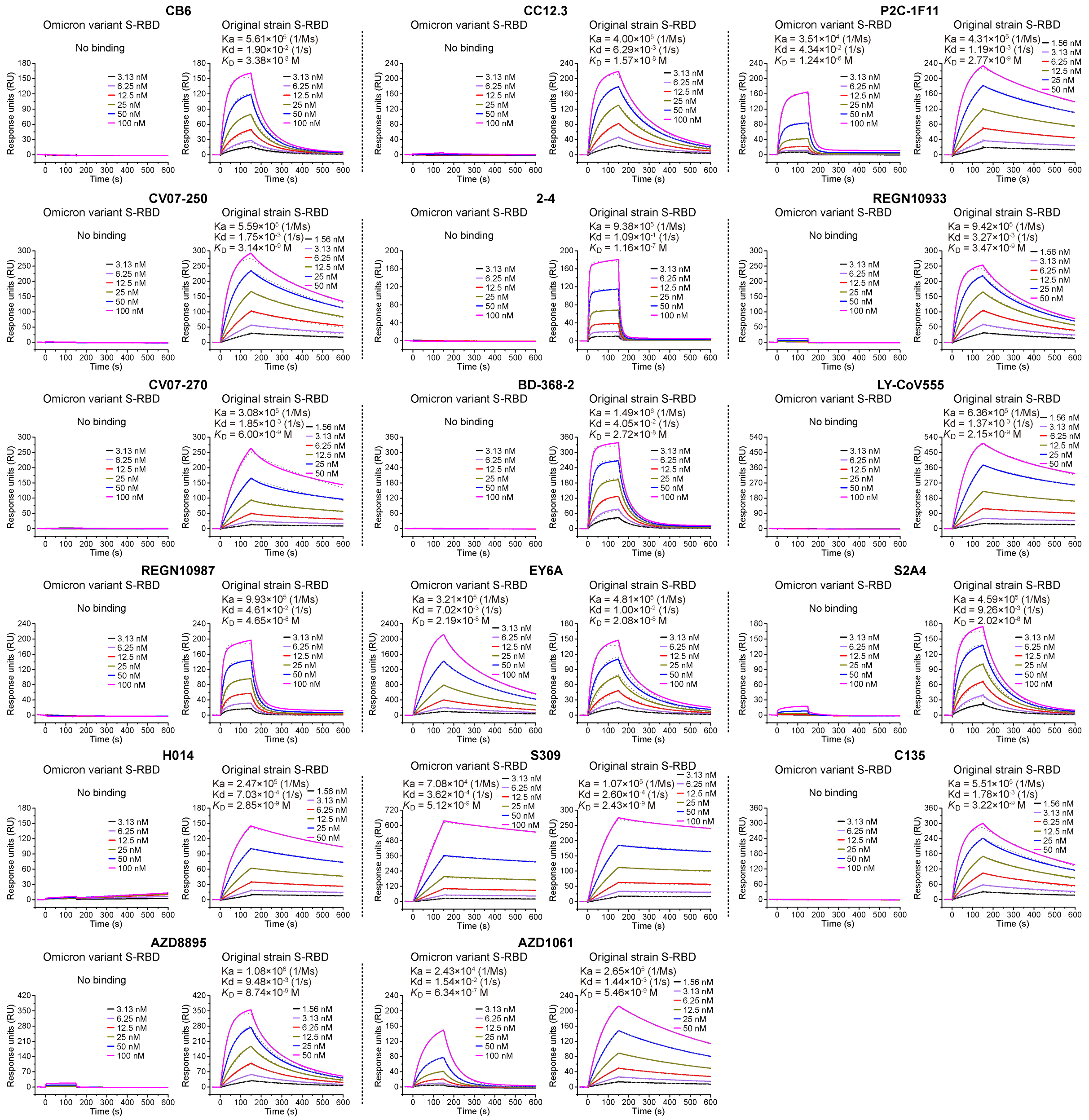


**Supplementary Fig. S2. An SPR assay characterizing the binding kinetics between** **SARS-CoV-2 S-RBD and indicated scFv antibody.** Gradient concentrations of scFv antibody (CB6, CC12.3, P2C-1F11, CV07-250, 2-4, REGN10933, CV07-270, BD-368-2, LY-CoV555, REGN10987, EY6A, S2A4, H014, S309, C135, AZD8895, or AZD1061) were flowed through SARS-CoV-2 S-RBD (original strain or Omicron variant) that immobilized on the sensor-chip surface. The real-time binding profiles and calculated kinetic parameters are shown.
